# Recurrent Red Eye Misdiagnosed as Conjunctivitis: Ocular Rosacea With Corneal Neovascularization in a Young Patient

**DOI:** 10.7759/cureus.108670

**Published:** 2026-05-11

**Authors:** Joana Ferreira, Aissatu Embalo, Ana Rodrigues, Sonia Cavaco, Gisela Ferreira

**Affiliations:** 1 General and Family Medicine, USF Luz do Tejo, ULS Arco Ribeirinho, Alcochete, PRT; 2 General and Family Medicine, UCSP Covelo, ULS São João, Porto, PRT

**Keywords:** blepharitis, conjunctivitis, corneal neovascularization, ocular rosacea, red eye

## Abstract

Chronic red eye is a prevalent presentation in both primary care and emergency settings. While most cases are self-limiting, recurrent or treatment-resistant episodes warrant systematic evaluation to exclude underlying chronic inflammatory conditions.

We report the case of a 19-year-old female patient with a longstanding history of bilateral red eye associated with pain, photophobia, and epiphora, initially and repeatedly managed as infectious conjunctivitis in the emergency department (ED). Despite multiple antibiotic courses, symptoms persisted. Ophthalmologic evaluation ultimately revealed chronic anterior and posterior blepharitis with meibomian gland dysfunction, peripheral corneal infiltrates, and neovascularization (NV) - findings consistent with ocular rosacea. Notably, subtle cutaneous signs of rosacea were also identified on clinical examination, further supporting the diagnosis. Following targeted therapy with oral doxycycline 100 mg twice daily, topical corticosteroids (CSs), and ocular lubricants, the patient achieved significant clinical improvement.

This case highlights the diagnostic pitfalls of recurrent red eye in primary care, the importance of recognizing chronic inflammatory ocular surface disease, and the value of timely ophthalmologic referral in preventing irreversible corneal complications.

## Introduction

Red eye is among the most frequent complaints in primary care and emergency settings, with the vast majority of cases attributed to self-limiting infectious or allergic conjunctivitis. However, recurrent or treatment-resistant presentations demand a more systematic diagnostic approach, as they may reflect underlying chronic inflammatory conditions such as blepharitis or ocular rosacea. Key clinical red flags that should prompt reconsideration of an infectious etiology include bilateral involvement, photophobia, pain, a known history of blepharitis, and failure to respond to two or more courses of topical antibiotics [1,2].

Ocular rosacea is a chronic inflammatory disorder affecting the ocular surface, frequently associated with cutaneous rosacea but capable of presenting in isolation. Up to 72% of patients with cutaneous rosacea will develop ocular involvement, either before or after the onset of skin disease [3]. Epidemiological studies estimate ocular manifestations occur in 6-72% of patients with rosacea, reflecting variation in diagnostic criteria and case ascertainment across studies [1,2]. Ocular manifestations may precede cutaneous findings by years, and in younger patients the absence of overt facial rosacea may further delay recognition [1,2].

The pathophysiology of ocular rosacea involves dysregulation of both the innate immune and neurovascular systems, with toll-like receptor activation and complement system involvement leading to chronic ocular surface inflammation. Alterations in the ocular microbiome have also been implicated in disease progression [4]. The pathophysiology is not entirely understood, but a multifactorial etiology is suspected, with both genetic and environmental factors contributing to its development and progression [2].

The 2017 updated classification by the National Rosacea Society Expert Committee introduced a phenotype-based approach, replacing the previous subtype system. This framework groups pathognomonic centrofacial erythema with major phenotypic criteria - papules, pustules, flushing, telangiectasia, and ocular involvement - with secondary phenotypes including burning, stinging, oedema, and dry appearance. Ocular involvement is recognised as a major diagnostic criterion within this framework [2].

Clinical features of ocular rosacea encompass a spectrum from mild to vision-threatening disease, including meibomian gland disease, blepharitis, conjunctivitis, peripheral corneal infiltrates, neovascularization (NV), and, rarely, corneal ulceration or perforation [4-6]. The differential diagnosis of recurrent red eye includes allergic conjunctivitis, infectious keratoconjunctivitis, anterior uveitis, dry eye disease, staphylococcal marginal keratitis, herpetic keratitis, and, in younger patients, blepharokeratoconjunctivitis (BKC) [1,5].

Ocular rosacea is of particular relevance in the pediatric and young adult population. In children, ocular rosacea may be misdiagnosed as viral or bacterial infections; unlike in adults, associated cutaneous changes are uncommon, and most disease is bilateral, though involvement may be asymmetric [7]. This underscores the importance of considering ocular rosacea in the differential diagnosis of recurrent red eye even in the absence of facial skin findings.

Management requires a stepwise, multidisciplinary approach incorporating ocular and skin hygiene, lifestyle modifications, and pharmacological interventions [4]. Doxycycline remains a cornerstone of systemic treatment, with therapeutic benefit extending beyond its antimicrobial properties through inhibition of matrix metalloproteinases and reduction of pro-inflammatory cytokines [5,8]. Early ophthalmologic referral is critical to prevent irreversible corneal complications [5,6].

This case illustrates the clinical challenge of distinguishing recurrent infectious conjunctivitis from chronic ocular surface inflammatory disease, and underscores the critical role of primary care physicians in recognizing patterns that warrant specialist referral.

## Case presentation

A 19-year-old female patient presented to primary care with a history of bilateral red eye dating from July 2025, associated with pain, burning sensation, foreign body sensation, photophobia, and persistent epiphora. She reported no significant medical history apart from blepharitis since the age of 11. She was not taking any regular medication and had no known allergies. There was no prior formal diagnosis of cutaneous rosacea, atopy, or autoimmune disease.

Her first emergency department (ED) attendance was on 25 July 2025, with a five-day history of right eye redness and burning of progressive onset. She was empirically treated with topical chloramphenicol eye drops and ointment, with a presumed diagnosis of bacterial conjunctivitis.

On 14 September 2025, she re-presented to the ED with recurrence of bilateral symptoms and was treated with topical azithromycin 1.5% eye drops. No microbiological investigations were performed at either attendance.

At the primary care assessment in October 2025, the following differential diagnoses were systematically considered and evaluated.

Bacterial conjunctivitis had been the working diagnosis at both ED attendances. However, three months of recurrent bilateral symptoms, the absence of mucopurulent discharge, and failure to respond to two courses of topical antibiotics (chloramphenicol and azithromycin) made this diagnosis untenable. No microbiological investigations were performed at either attendance, which is acknowledged as a limitation; however, the overall clinical pattern was not consistent with acute infectious disease.

Chlamydial conjunctivitis was considered given the chronicity and bilateral involvement. However, the patient had no identified risk factors for sexually transmitted infection, no follicular conjunctival reaction was documented, and the pattern of recurrence with photophobia and blepharitis was not consistent with chlamydial disease.

Allergic or vernal keratoconjunctivitis was considered given the bilateral presentation and epiphora. However, the predominance of pain and photophobia over pruritus, the absence of personal or family history of atopy, and the absence of giant papillae argued against this diagnosis.

Anterior uveitis was considered given the photophobia and ocular pain. However, the absence of anterior chamber reaction - confirmed by a negative Tyndall effect at ophthalmologic evaluation - and preserved visual acuity (VA) made this diagnosis unlikely.

Herpetic keratitis was considered given the recurrent nature of symptoms and corneal involvement. However, the bilateral presentation, absence of dendritic epithelial lesions on fluorescein staining (negative at ophthalmologic evaluation), and absence of prior herpetic episodes or immunosuppression argued against this diagnosis.

Staphylococcal marginal keratitis was considered given the peripheral corneal infiltrates identified at ophthalmologic evaluation. This condition, caused by immune-mediated hypersensitivity to Staphylococcal exotoxins, typically presents with peripheral corneal infiltrates with a clear interval from the limbus. While this diagnosis cannot be entirely excluded, the longstanding history of blepharitis since childhood, bilateral recurrence, and sustained response to anti-inflammatory therapy targeting ocular rosacea supported the latter as the primary diagnosis.

Contact lens-related keratitis was excluded as the patient had no history of contact lens wear.

Dry-eye disease was considered given the burning, foreign body sensation, and epiphora. However, the degree of conjunctival hyperemia, the history of blepharitis since childhood, and the progressive corneal involvement were not consistent with isolated dry eye disease.

Autoimmune peripheral keratitis, as seen in rheumatoid arthritis, systemic lupus erythematosus, or granulomatosis with polyangiitis, was considered given the peripheral location of corneal infiltrates and NV. However, the patient had no history of systemic autoimmune disease, no relevant systemic symptoms, and the overall clinical picture - with longstanding blepharitis and meibomian gland dysfunction as the primary ocular surface disease - was more consistent with ocular rosacea.

The combination of longstanding bilateral blepharitis since childhood, recurrent photophobia, bilateral conjunctival hyperemia, peripheral corneal infiltrates and NV at the 3 and 9 o'clock positions, treatment resistance to topical antibiotics, and the identification of subtle cutaneous rosacea signs was considered highly consistent with ocular rosacea, further supported by the sustained clinical response to targeted anti-inflammatory therapy.

Following a third episode in October 2025, after approximately three months of recurrent and incompletely resolving symptoms including discomfort, photophobia, bilateral redness, and pain, she was assessed in primary care. Given the chronicity and treatment resistance of her symptoms, oral doxycycline 100 mg twice daily was initiated alongside topical hydrocortisone acetate eye drops (Softacort) three times daily, ofloxacin eye drops, and sodium hyaluronate/ectoin lubricant eye drops for one month. She was subsequently referred for ophthalmologic assessment.

Ophthalmologic evaluation took place on 13 January 2026. Best-corrected visual acuity (BCVA) was 1.0 bilaterally. Manifest refraction revealed -0.75 / -0.50 x 15° in the right eye and -0.75 / -0.50 x 5° in the left eye.

**Figure 1:**
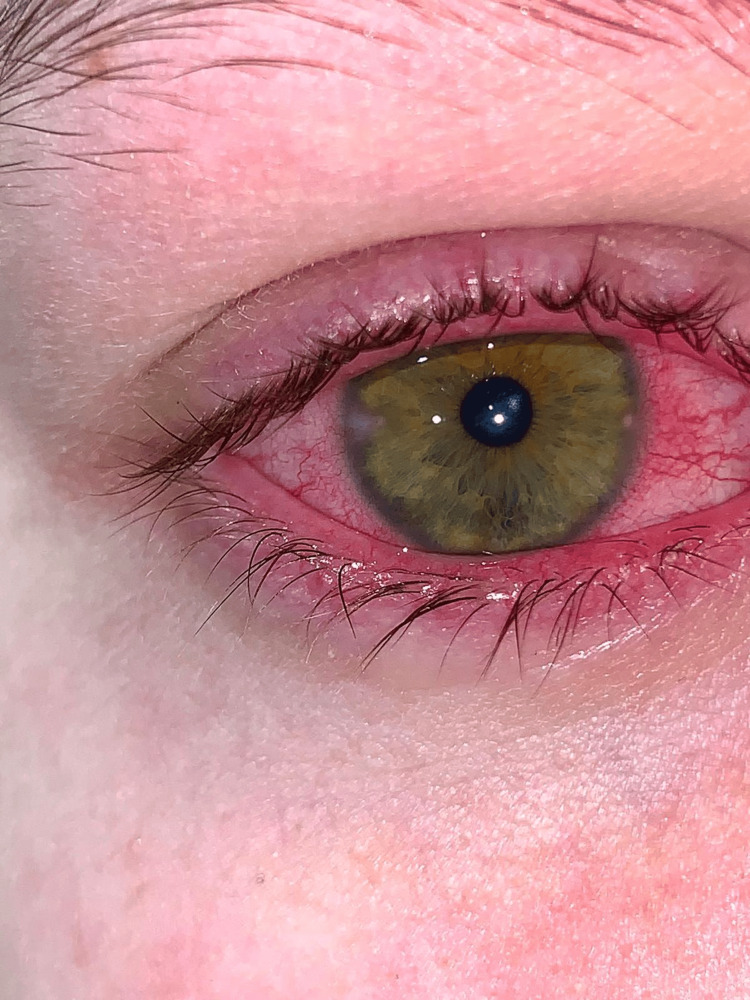
Slit-lamp photograph of the left eye demonstrating peripheral corneal NV and conjunctival hyperemia consistent with ocular rosacea. Clinical photograph of the left eye obtained with a conventional slit-lamp, demonstrating diffuse conjunctival hyperemia and peripheral corneal infiltrates with associated NV at the 3 and 9 o’clock positions (annotated with circles). No epithelial defects were identified on fluorescein staining. These findings are consistent with chronic ocular surface inflammation in the context of posterior blepharitis and meibomian gland dysfunction, in keeping with a diagnosis of ocular rosacea. Photograph taken approximately one month after initiation of oral doxycycline 100 mg twice daily and topical CS therapy in primary care, and prior to formal ophthalmologic evaluation. NV: Neovascularization; CS: Corticosteroid

Slit-lamp examination of the right eye demonstrated anterior and posterior blepharitis with meibomian gland dysfunction, marked conjunctival hyperemia, and peripheral corneal NV at the 3 and 9 o’clock positions. No anterior chamber reaction was identified (Tyndall effect absent). Examination of the left eye revealed diffuse conjunctival hyperemia, peripheral corneal infiltrates, and significant NV at the 3 and 9 o’clock positions. No epithelial defects were identified on fluorescein staining in either eye. Findings were consistent with chronic ocular surface inflammation secondary to meibomian gland dysfunction and blepharitis (Figure 1).

The overall clinical picture - chronic blepharitis, meibomian gland dysfunction, peripheral corneal infiltrates and NV, and the longstanding recurrent nature of symptoms since adolescence - was considered highly consistent with ocular rosacea. The patient was referred to dermatology for evaluation of potential cutaneous rosacea and further characterization of the systemic disease.

The treatment regimen was updated to: topical dexamethasone (Dexafree) every 8h + Predniftalmina + oral doxycycline 100mg BD (continued) + Hylo Dual lubricant eye drops, targeting both the inflammatory component and the underlying meibomian gland dysfunction. Eyelid hygiene measures were also reinforced.

At follow-up over a period of approximately five months (November 2025 to April 2026), the patient maintained substantial clinical improvement on the current treatment regimen. Symptoms of redness, pain, and photophobia remain well controlled, with no further ED attendances for red eye. VA has remained stable at 1.0 bilaterally. Oral doxycycline 100 mg twice daily has been maintained given the sustained clinical response and the severity of corneal involvement. Dermatological evaluation is scheduled and awaited for comprehensive management of the systemic disease.

**Table 1:** Clinical timeline of symptom onset, ED attendances, primary care assessment, ophthalmologic evaluation, and follow-up GFM: General and family medicine; BCVA: Best-corrected visual acuity; ED: Emergency department; NV: Neovascularization; VA: Visual acuity

Date	Setting	Symptoms / Findings	Management
July 2025	—	Onset of bilateral red eye with pain, burning sensation, foreign body sensation, photophobia, and persistent epiphora	—
July 2025	ED	Right eye redness and burning, 5 days, progressive worsening	Topical chloramphenicol eye drops and ointment (presumed bacterial conjunctivitis)
September 2025	ED	Recurrence of bilateral symptoms	Topical azithromycin 1.5% eye drops
October 2025	Primary Care (GFM)	3 months of recurrent bilateral red eye, pain, photophobia; treatment-resistant	Oral doxycycline 100 mg twice daily + topical hydrocortisone acetate (Softacort) three times daily + ofloxacin eye drops (Floxedol) + Hylo Dual lubricant eye drops for one month; referred to ophthalmology
January 2026	Ophthalmology	BCVA 1.0 bilaterally; anterior and posterior blepharitis; marked conjunctival hyperemia; peripheral corneal NV at 3 and 9 o'clock (both eyes); peripheral corneal infiltrates (left eye); Tyndall negative; fluorescein staining negative	Topical dexamethasone (Dexafree) every 8 hours + oral doxycycline 100 mg twice daily (continued) + Hylo Dual lubricant eye drops + Predniftalmina eye drops
November 2025-April 2026	Follow-Up	Sustained clinical improvement; no further ED attendances; VA stable 1.0 bilaterally	Current regimen maintained

## Discussion

This case illustrates a clinically instructive pattern: a young patient with recurrent red eye repeatedly managed as infectious conjunctivitis, in whom delayed recognition of an underlying chronic inflammatory condition led to corneal complications. Three ED attendances over three months, each resulting in empirical antibiotic treatment, underscore the diagnostic challenge posed by early ocular rosacea in primary care.

Blepharitis is a common chronic inflammatory condition of the eyelid margins, classified as anterior (affecting the lash follicles, commonly associated with Staphylococcus colonization) or posterior (affecting the meibomian glands). Posterior blepharitis, or meibomian gland dysfunction, disrupts lipid secretion, destabilizes the tear film, and promotes ocular surface inflammation. When chronic and inadequately treated, this inflammatory milieu can drive peripheral corneal infiltration and NV - findings that, in this patient, reflected years of undertreated disease beginning in childhood [5,6].

Ocular rosacea is an important and frequently overlooked cause of chronic blepharitis, particularly in younger patients. It is characterized by recurrent blepharitis, meibomian gland dysfunction, conjunctival hyperemia, and, in advanced cases, corneal involvement including peripheral infiltrates, vascularization, and, rarely, ulceration. Crucially, ocular manifestations may precede or occur independently of cutaneous findings, though in this case subtle facial erythema and telangiectasias were also identified, corroborating the diagnosis. This highlights the importance of a thorough general examination in patients presenting with chronic ocular surface disease [1,2].

Corneal NV, as observed in this patient, is a particularly significant finding. Physiologically, the cornea is avascular; persistent inflammation triggers the release of pro-angiogenic mediators, including vascular endothelial growth factor (VEGF), promoting neovascular ingrowth from the limbal vasculature. Once established, corneal NV is largely irreversible and may compromise VA, optical clarity, and the feasibility of future corneal interventions such as transplantation or contact lens wear [9].

Doxycycline remains a cornerstone of treatment for ocular rosacea. Its therapeutic benefit extends beyond its antimicrobial properties: at sub-antimicrobial doses, doxycycline inhibits matrix metalloproteinases, reduces pro-inflammatory cytokine production, and suppresses meibomian gland lipase activity, thereby improving meibum quality and reducing ocular surface inflammation. The sustained clinical improvement observed in this patient over five months of doxycycline 100 mg twice daily is consistent with its established anti-inflammatory mechanism of action and strongly supports the diagnosis of ocular rosacea. Although lower sub-antimicrobial doses (50 mg once daily) have been proposed for long-term maintenance, the severity of corneal involvement in this case justified continuation of the full anti-inflammatory dose under specialist guidance [5,8].

The therapeutic approach adopted in this case merits explicit justification, particularly given the primary care setting in which it was initiated. Following three months of recurrent, treatment-resistant symptoms and two failed courses of topical antibiotics, the clinical picture was considered inconsistent with infectious conjunctivitis and prompted empirical treatment targeting chronic ocular surface inflammation, pending ophthalmologic evaluation.

Oral doxycycline 100 mg twice daily was selected as the systemic agent of choice. Its therapeutic benefit in ocular rosacea and meibomian gland dysfunction extends beyond antimicrobial activity: at anti-inflammatory doses, doxycycline inhibits matrix metalloproteinases, suppresses pro-inflammatory cytokine production, and reduces meibomian gland lipase activity, thereby improving meibum quality and reducing ocular surface inflammation [5,8]. The full anti-inflammatory dose of 100 mg twice daily was maintained throughout, given the severity of corneal involvement identified at subsequent ophthalmologic evaluation. Although sub-antimicrobial doses (50 mg once daily) have been proposed for long-term maintenance, the degree of corneal NV in this case justified continuation of the higher dose under specialist guidance [5,8].

Topical hydrocortisone acetate (Softacort) three times daily was initiated in primary care to reduce acute ocular surface inflammation. Short-term topical corticosteroids (CSs) are an accepted adjunct in the management of ocular rosacea exacerbations, particularly when conjunctival hyperemia and corneal infiltrates are present [5]. Their use was subsequently reviewed and adjusted at ophthalmologic evaluation, where topical dexamethasone (Dexafree) every eight hours and prednisolone-based eye drops (Predniftalmina) were prescribed, reflecting the severity of findings on slit-lamp examination. CS use was maintained under ophthalmologic supervision given the extent of corneal involvement.

Ofloxacin eye drops (Floxedol) were included in the initial primary care regimen to provide additional cover against secondary bacterial colonization of the lid margin, a recognized contributor to the inflammatory cascade in posterior blepharitis [5,6]. Preservative-free sodium hyaluronate/ectoin lubricant eye drops (Hylo Dual) were prescribed to support tear film stability and alleviate symptoms of meibomian gland dysfunction, consistent with current stepwise management recommendations [5,6].

Eyelid hygiene measures, including warm compresses and lid margin cleansing, were reinforced at both primary care and ophthalmology appointments, as these remain a fundamental non-pharmacological component of blepharitis and meibomian gland dysfunction management [4].

The initiation of this regimen in primary care, prior to ophthalmologic evaluation, reflects a pragmatic approach in a patient with a prolonged history of treatment-resistant symptoms. The sustained clinical improvement observed over five months of follow-up supports the appropriateness of the therapeutic strategy and the underlying inflammatory etiology.

From a primary care perspective, this case highlights several actionable learning points. Firstly, recurrent red eye that does not resolve with two or more courses of topical antibiotics should prompt reconsideration of the diagnosis. Secondly, a history of blepharitis since childhood, combined with recurrent episodes of photophobia and bilateral involvement, should raise suspicion for a chronic inflammatory etiology. Thirdly, early ophthalmologic referral is essential: in this case, ophthalmologic assessment at the second or third episode may have prevented the development of corneal NV. Finally, ocular rosacea should be considered in the differential diagnosis of recurrent blepharitis even in the absence of facial rosacea, particularly in young patients.

**Table 2:** Comparison of the present case with selected published cases and recent literature on ocular rosacea, including patient characteristics, clinical features, diagnostic delay, treatment, and outcomes BKC: Blepharokeratoconjunctivitis; CS: Corticosteroid; ED: Emergency department; NOS: Not otherwise specified; NV: Neovascularization; VA: Visual acuity; OE: Left eye

Feature	Present case	Putri MP, Naryati LP [8]	Arman et al. [10]	Quarterman et al. [11]	Mohamed-Noriega et al. [4]
Age / Sex	19F	12M	Adults (mean 43)	Adults (mean 43)	Adults + children
Initial misdiagnosis	Bacterial conjunctivitis (×2 ED)	Recurrent hordeolum / conjunctivitis	Blepharitis NOS	Not specified	Conjunctivitis / dry eye / blepharitis
Diagnostic delay	~3 months	Since childhood	Months–years	Not specified	Years (especially children)
Cutaneous rosacea	Subtle (dermatology pending)	Nasal papulopustular lesions	Present in most	Present in all	Absent in up to 80% of pediatric cases
Corneal involvement	Bilateral NV at 3+9h; peripheral infiltrates OE	Unilateral NV + stromal scar	NV 50%; infiltrates 17%	Not described	NV, infiltrates, ulceration, perforation
Treatment	Doxycycline 100mg BD + topical CS + ofloxacin + lubricants	Doxycycline + topical CS + lubricants	Doxycycline vs topical cyclosporine	Doxycycline 100mg BD	Stepwise: hygiene + doxycycline + topical CS ± cyclosporine
Outcome	Improved at 5 months; VA 1.0 bilaterally	Improved VA and symptoms	Significant improvement both arms	Significant symptom reduction	Variable; irreversible NV if delayed

The present case shares several features with published reports of ocular rosacea, including the recurrent nature of symptoms, initial misdiagnosis as infectious conjunctivitis, and favorable response to doxycycline-based anti-inflammatory therapy. A distinctive feature is the young age at presentation (19 years) and early onset of blepharitis (since age 11), consistent with the pediatric form of ocular rosacea - BKC - in which cutaneous findings are frequently absent, further complicating and delaying diagnosis [7]. The bilateral corneal NV at the 3 and 9 o'clock positions reflects chronic limbal ischaemia secondary to prolonged ocular surface inflammation, and underscores the visual risk associated with delayed recognition and referral [4].

Several limitations of this case report should be acknowledged. First, the diagnosis of ocular rosacea, while strongly supported by the clinical picture, ophthalmologic findings, and sustained response to targeted anti-inflammatory therapy, remains pending formal dermatological confirmation. Dermatological evaluation has been scheduled and is awaited; until then, the diagnosis should be regarded as highly consistent with, rather than definitively confirmed as, ocular rosacea.

Second, microbiological investigations were not performed at either ED attendance, which precludes definitive exclusion of an infectious etiology at initial presentation. While the overall clinical pattern was not consistent with acute infectious conjunctivitis, the absence of cultures or polymerase chain reaction (PCR) testing represents a methodological limitation.

Third, objective quantification of the extent of corneal NV at baseline and during follow-up was not available from the clinical record. More granular data on NV progression or regression under treatment would have strengthened the reporting of clinical outcomes. VA remained stable at 1.0 bilaterally throughout follow-up, providing an indirect measure of preserved corneal function.

Fourth, before-and-after slit-lamp photographs would have provided more compelling documentation of the clinical response to treatment. A post-treatment photograph of equivalent quality suitable for publication is not available. The picture from Figure 1 was obtained approximately one month after initiation of primary care treatment and prior to formal ophthalmologic evaluation.

Finally, as a single case report, no generalizable conclusions can be drawn regarding the prevalence of ocular rosacea misdiagnosis in primary care, nor regarding the comparative efficacy of the treatment regimen used. The case is intended to illustrate a clinically instructive diagnostic pattern rather than to establish evidence-based recommendations.

## Conclusions

Recurrent or treatment-resistant red eye should prompt systematic evaluation for underlying chronic inflammatory conditions, including blepharitis and ocular rosacea. This case demonstrates how repeated empirical antibiotic treatment of presumed infectious conjunctivitis can delay the recognition of chronic ocular surface disease and allow progression to corneal NV. The sustained clinical response to targeted anti-inflammatory therapy over five months further supports the underlying inflammatory etiology. Primary care physicians should maintain a low threshold for ophthalmologic referral in patients with recurrent, bilateral, or photophobia-associated red eye, particularly when symptoms persist beyond two treatment courses. Early diagnosis and targeted anti-inflammatory therapy are essential to prevent irreversible corneal sequelae and preserve long-term visual outcomes.

As formal dermatological evaluation remains pending, the diagnosis of ocular rosacea should be regarded as highly consistent with the overall clinical picture, pending definitive confirmation.
